# Machine learning-based quantitative prediction of drug exposure in drug-drug interactions using drug label information

**DOI:** 10.1038/s41746-022-00639-0

**Published:** 2022-07-11

**Authors:** Ha Young Jang, Jihyeon Song, Jae Hyun Kim, Howard Lee, In-Wha Kim, Bongki Moon, Jung Mi Oh

**Affiliations:** 1 College of Pharmacy and Research Institute of Pharmaceutical Sciences, Seoul National University, Seoul, Republic of Korea; 2 Department of Computer Science and Engineering, Seoul National University, Seoul, Republic of Korea; 3 School of Pharmacy, Jeonbuk National University, Jeonju, Republic of Korea; 4 Department of Clinical Pharmacology and Therapeutics, Seoul National University College of Medicine and Hospital, Seoul, Korea

**Keywords:** Outcomes research, Preclinical research, Machine learning

## Abstract

Many machine learning techniques provide a simple prediction for drug-drug interactions (DDIs). However, a systematically constructed database with pharmacokinetic (PK) DDI information does not exist, nor is there a machine learning model that numerically predicts PK fold change (FC) with it. Therefore, we propose a PK DDI prediction (PK-DDIP) model for quantitative DDI prediction with high accuracy, while constructing a highly reliable PK-DDI database. Reliable information of 3,627 PK DDIs was constructed from 3,587 drugs using 38,711 Food and Drug Administration (FDA) drug labels. This PK-DDIP model predicted the FC of the area under the time-concentration curve (AUC) within ± 0.5959. The prediction proportions within 0.8–1.25-fold, 0.67–1.5-fold, and 0.5–2-fold of the AUC were 75.77, 86.68, and 94.76%, respectively. Two external validations confirmed good prediction performance for newly updated FDA labels and FC from patients’. This model enables potential DDI evaluation before clinical trials, which will save time and cost.

## Introduction

A drug-drug interaction (DDI) occurs when the pharmacokinetics (PK) or pharmacodynamics (PD) of the victim drug is changed by a perpetrator drug previously taken or administered in combination. DDIs may lead to products’ withdrawal from the market. For instance, astemizole, a drug for the treatment of allergic symptoms, was withdrawn from the market due to the possibility of prolongation of the QT interval and arrhythmias when combined with cytochrome P450 3A4 (CYP3A4) inhibitors, including grapefruit juice and erythromycin^1^. Mibefradil, a treatment for hypertension and chronic angina, was withdrawn from the market due to bradycardia and rhabdomyolysis when combined with various cardiovascular drugs, such as beta-blockers or statins^2^. Likewise, DDIs have been studied as one of the causes of severe adverse reactions occurring in clinical settings^3,4^. Furthermore, the increasing trend of multi-drug prescriptions increases the possibility of side effects due to DDIs^5^.

However, despite this importance, numerous DDIs exist, but have not been identified. What is worse, approximately 10% of DDI pairs may have adverse reactions due to DDIs among all combinations of commercially available drugs^6^. This is because, first, the Food and Drug Administration (FDA) recommends that a clinical trial for DDIs be conducted when drugs affect only, or are affected by, a specific enzyme in an in-vitro study^7^. High costs and time-consuming clinical trials may be part of the reason for the limited number of known DDIs. Second, the mechanisms by which DDIs occur are very diverse, and each mechanism may be complex, so not all potential DDIs may be detected.

Various machine learning techniques have been developed to predict DDIs to overcome the lack of known DDI pairs. In previous studies^8–42^, many models have been developed to predict the presence or absence of DDIs, discovering DDI pairs that cause side effects, or classifying the types of DDIs using open source databases (DBs). However, there are clear limitations. First, most models have only provided a simple prediction for the existence or classification of DDIs. These models do not aid in complex clinical decisions, such as precise dose adjustment or alternative drug selection. Predictions about fold change of PK parameters are needed to help physicians and pharmacists, but, to date, there are no models that have been successful in predicting this. Second, a systematically constructed true-negative dataset does not exist. The DDI DB, such as DrugBank, widely used for DDIs prediction, contains information that ‘there is a DDI between drug A and B’, but does not contain information that ‘there is no DDI’. As a result, researchers inevitably have selected random sets of drug pairs thinking there were no interactions^9,11,19,20,28–32,34,37,42,43^. Certainly, absence of evidence is not evidence of absence. Using a model without good input makes it difficult to obtain reliable output. If the negative set is random, it is difficult to identify the exact cause when unexpected problematic output occurs.

**Fig. 1:**
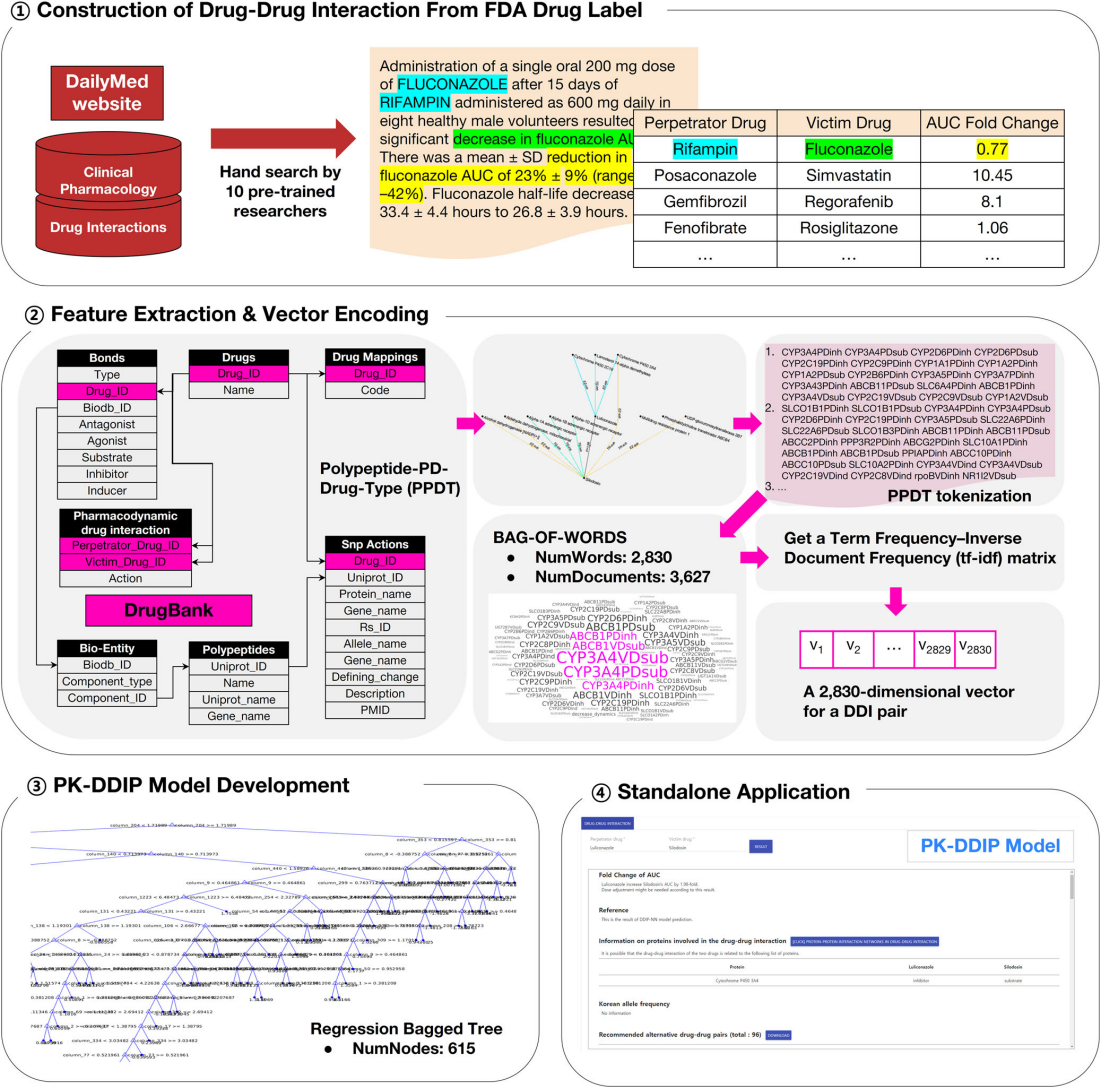
Drug-drug interaction (DDI) Prediction Pipeline Overview. (Step 1) Reliable Food and Drug Administration (FDA) drug labels were used through the DailyMed website to build the pharmacokinetic (PK)-DDI dataset. A total of 38,711 FDA drug labels were obtained (Evaluation date: May 2020) from sentences/pictures/tables in the clinical pharmacology and drug interaction sections. (Step 2) Information on various drug properties from DrugBank (Evaluation date: March 2021) was obtained. Drug properties data may be arranged around the perpetrator and the victim drugs, and various polypeptides are radially linked. Polypeptide-PD (pharmacodynamics)-Drug-Type (PPDT) tokenization was proposed to represent drug pairs. A bag-of-words containing 2830 unique tokens was obtained. Each drug-drug pair was encoded as a 2830-dimensional vector through normalization with a Term Frequency-Inverse Document Frequency (tf-idf) matrix of bag-of-words. (Step 3) The Bagged (Bootstrap Aggregation) Tree method was used as an application model. The tree consisted of 615 branches and had 308 nodes for which fold change values were determined. (Step 4) A standalone application PK-DDI prediction (PK-DDIP) model is provided. Through this application, users may obtain predicted and reported fold change values, drug polypeptide information and its plot, single-nucleotide polymorphisms action, and alternative drug recommendation information at the 4^th^ anatomical therapeutic chemical level.

Therefore, a sufficient amount of DDI information containing fold change of PK parameters was collected by hand search from FDA drug labels for high model performance and a reliable PK-DDI DB was constructed (Fig. 1). Using this data, a PK DDI prediction model (PK-DDIP model) is proposed that quantitatively predicts the fold change of drug PK parameters in DDIs. In addition, a standalone application, which provides predicted fold changes and reported fold changes of PK parameters, anatomical therapeutic chemical (ATC) code-based alternative drug choices, and single nucleotide polymorphism (SNP) action information was distributed.

## Results

### Construction of the PK-DDI DB

A total of 38,711 FDA drug label data for 3,587 prescription drugs was downloaded in an XML format from the DailyMed website. After applying standard operating procedures, 3,627 reliable DDI information as a training dataset for PK-DDIP model, including area under the time-concentration curve (AUC) fold change values, were selected (Supplementary Fig. 1). Of the 3,627 DDIs, 1,189 were positive (765: increase; 424: decrease), and 2,438 were negative. The median fold change value for the AUC was 1.82 (interquartile range (IQR) 1.45–2.8; min-max 1.26-190), 0.55 (IQR 0.31–0.69; min-max 0.03-0.8), and 1.0 (IQR 1.0–1.0; min-max 0.81-1.25) for categories of increase, decrease, and negative, respectively.

### Model performance

**Fig. 2:**
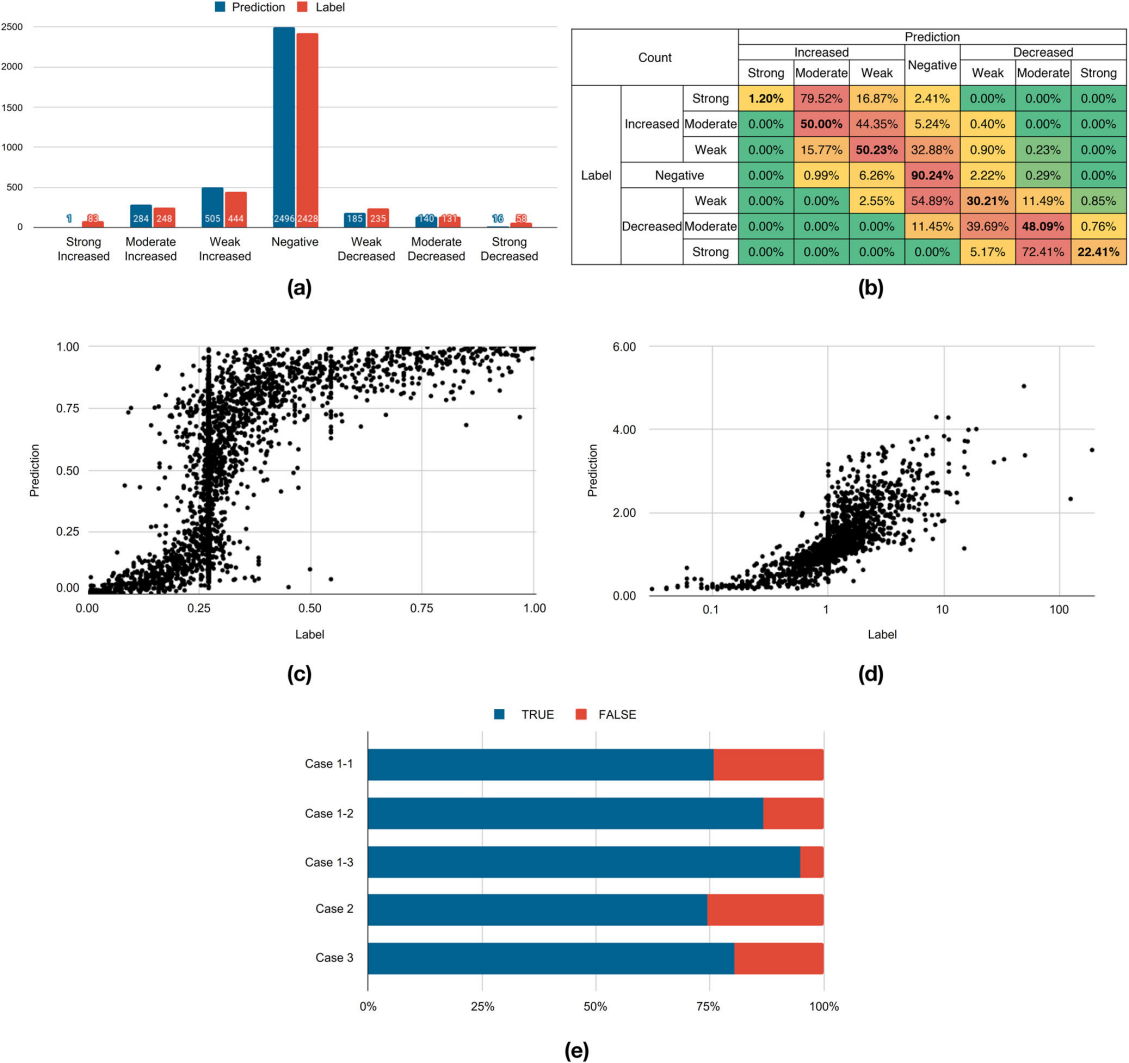
Model performance. **a** Distribution of predicted and labeled drug-drug interactions (DDIs) according to the Food and Drug Administration’s (FDA) classification criteria. A strong DDI means that the perpetrator drug increases the area under the time-concentration curve (AUC) of the victim drug by more than 5-fold or decreases the AUC to less than 0.2-fold. In moderate DDI, the perpetrator increases the victim drug AUC by 2- to 5-fold or decreases the victim drug AUC by 0.2 to 0.5-fold. When weak DDI occurs, the perpetrator increases the victim drug AUC by 1.25- to 2-fold or decreases it by 0.5- to 0.8-fold. The AUC fold change (FC) between 0.8- and 1.25-fold, which does not belong to any criteria, is defined as a negative DDI. **b** Heatmap for the predicted percentage of DDI classes correctly called among each DDI class in the label. Cells with higher percentages are colored red for each prediction class. **c** Percentage rank of a given value in a data set. **d** Scatter chart. **e** Evaluation for quantitative AUC FC. Case 1-1: {0.8 × *FC*_*lab*_ ≤ *FC*_*pre*_} ∧ {1.25 × *FC*_*lab*_ ≥ *FC*_*pre*_}. Case 1-2: {0.67 × *FC*_*lab*_ ≤ *FC*_*pre*_} ∧ {1.5 × *FC*_*lab*_ ≥ *FC*_*pre*_}. Case 1-3: {0.5 × *FC*_*lab*_ ≤ *FC*_*pre*_} ∧ {2 × *FC*_*lab*_ ≥ *FC*_*pre*_}. Case 2: *Class*_*lab*_ = *Class*_*pre*_. Case 3: Case 1-1 ∨ Case 2. Lab, Label from FDA; Pre, Prediction from DDI prediction.

Figure 2 is the evaluation of the PK-DDIP model against various criteria. Figure 2a shows the DDI class distribution of predicted fold changes and label fold changes of victim drug AUCs. The distribution of DDI class predicted by the model was generally very similar to the DDI class distribution of the label. However, the values predicted by the model were less distributed for a strong DDI class.

Figure 2b more explicitly shows which class the PK-DDIP model predicts. Among the seven classes, classes with a moderate increase, moderate decrease, negative predictions, and a weak increase were accurately predicted. Among them, the prediction accuracy of a negative prediction was 90.2%. In the case of the strong decrease class, although only 22.4% of the pairs were predicted as the correct class, 72.4% of the drug pairs were predicted as the moderate decrease class. However, similar to the problem in Fig. 2a, for the strong increase class, it was confirmed that only 1.2% of the strong increase classes could be correctly predicted, but 79.5% were predicted as the moderate increase class.

The model obtained a 5-fold cross-validation result of a root-mean-squared error (RMSE) of 0.5959 (Supplementary Table 1) and predicted the fold change within 0.5959 on average (Fig. 2c, Fig. 2d). Median percent error of the predicted fold change from the label was 7.9%, with an IQR of 2.4–23.9%. When the PK fold change value was at an extreme level such as the strong increase or strong decrease category, the percent error value tended to increase, whereas in negative or weak increase/decrease, the percent error value was small (Supplementary Table 2). However, this is only an average, the fold change range varies, and the fold change corresponding to a negative or weak class was significantly affected by even a tiny error. Therefore, numerical values were evaluated according to pre-specified DDI classification criteria. Figure 2e shows the accuracy according to five DDI classification criteria. Case 1-1 had the most rigorous evaluation where 75.77% of pairs entered the interval, followed by 86.68% for Case 1-2 and 94.76% for Case 1-3. The accuracy of predicting the exact class (Case 2) and achieving a combined task (Case 3) was 74.33% and 80.29%, respectively.

### External validation of model 1: Comparison with newly updated FDA labels

Newly reported DDI information was collected and compared with prediction results from the PK-DDIP model (Table 1). Twenty-one drug pairs contained new information on the change in the AUC of victim drugs. Among the 21 drug pairs, seven of ten DDIs in newly approved drugs were closely predicted and evaluated as ‘good’. Six of 11 newly updated drug labels and the remaining five were evaluated as good, and moderate, respectively. All 21 drug pairs achieved at least a ‘moderate’ grade, which meant that the PK-DDIP model satisfactorily predicted the DDI direction and extent.

**Table 1:** Comparison of newly updated drug interaction information with area under the time-concentration curve (AUC) fold changes in the label and predicted AUC fold changes by the pharmacokinetic drug-drug interaction prediction (PK-DDIP) model.

Perpetrator drugs	Victim drugs	AUC fold change from updated FDA label	AUC fold change from PK-DDIP model	Evaluation^1^
**Newly approved drugs**				
Gemfibrozil	Ozanimod	1.58	2.43	Moderate
Rifampin	Ozanimod	0.42	0.34	Good
Itraconazole	Pemigatinib	1.88	2.28	Good
Rifampin	Pemigatinib	0.15	0.32	Moderate
Esomeprazole	Pemigatinib	1	0.92	Good
Ranitidine	Pemigatinib	1	1.01	Good
Itraconazole	Ripretinib	1.99	2.09	Good
Rifampin	Ripretinib	0.39	0.36	Good
Efavirenz	Ripretinib	0.44	0.56	Moderate
Pantoprazole	Ripretinib	1	0.83	Good
**Drugs with updated drug labels**				
Cannabidiol	Stiripentol	1.42	1.52	Good
Rifampin	Darolutamide	0.28	0.33	Good
Itraconazole	Darolutamide	1.7	2.21	Moderate
Enasidenib	Rosuvastatin	3.44	1.35	Moderate
Enasidenib	Digoxin	1.22	1.16	Good
Cimetidine	Hydroxychloroquine	2	1.18	Moderate
Fluconazole	Macitentan	4	1.63	Moderate
Cabotegravir	Rilpivirine	0.99	1.02	Good
Rilpivirine	Cabotegravir	1.12	1.02	Good
Tafamidis	Midazolam	1	1.08	Good
Tafamidis	Rosuvastatin	1.96	1.17	Moderate

^1^If 0.8*fold change < machine learning prediction result < 1.25*fold change, it is judged as good. If only classification of whether AUC fold change is > 1 (or < 1) is successful, then the evaluation value is moderate. *FDA* Food and Drug Administration.

### External validation of model 2: Comparison with real patients’ results

**Fig. 3:**
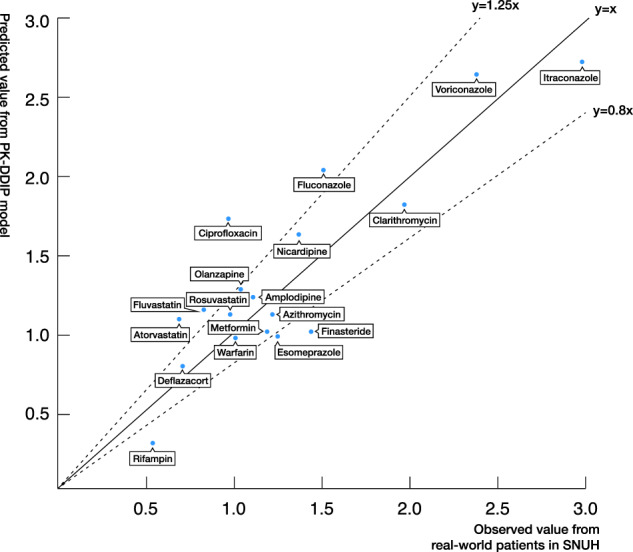
Comparison with real patients’ result. The comparison of pharmacokinetic drug-drug interaction prediction (PKDDIP) model results (predicted fold change values) and observed real-world patients’ results using tacrolimus as a victim drug in a tertiary hospital clinical data warehouse. SNUH, Seoul National University Hospital.

Data from 8,684 patients who used tacrolimus at a tertiary hospital were collected (Supplementary Fig. 2). After excluding patients without tacrolimus lab data, the 18 eligible cohorts included 2,143 patients. The value of correlation coefficient (R-squared) between predicted values and observed values was 76.9% (Fig. 3). The 11 of 18 DDI pairs were satisfactorily predicted except for the failure of the 7 pairs (atorvastatin, ciprofloxacin, esomeprazole, finasteride, fluconazole, fluvastatin, and rifampin). The significant differences between the two values were observed as follows. (Predicted value: observed value = 1.08:0.69 [atorvastatin], 1.72:0.9 [ciprofloxacin], 0.97:1.25 [esomeprazole], 1:1.44 [finasteride], 2.03:1.51 [fluconazole], 1.14:0.83 [fluvastatin], and 0.29:0.54 [rifampin])

### Standalone application

**Fig. 4:**
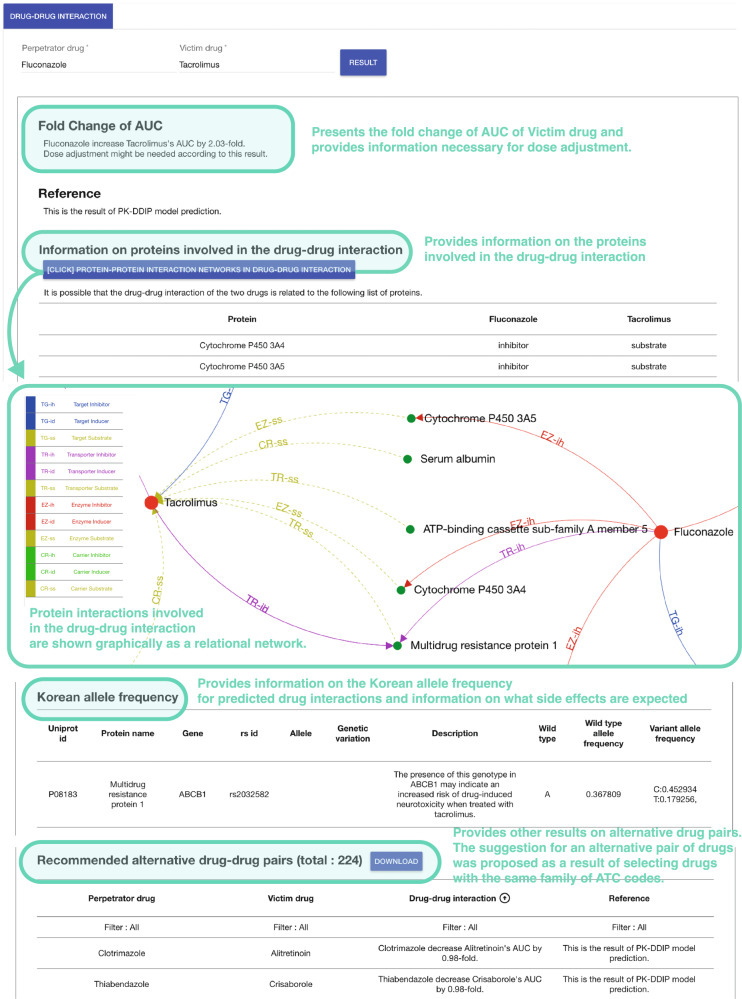
Standalone application. ATC anatomical therapeutic chemical, AUC area under the time-concentration curve, PK-DDIP pharmacokinetic drug-drug interaction prediction.

A standalone application provided the following features (Fig. 4). Users may search by entering perpetrator and victim drugs. In a protein-protein interaction network, the application helps the user identify the fold change and polypeptide bond relationship between the perpetrator drug and the victim drug, which can also be shown graphically as a relational network. The application also provides SNP action information of the entered perpetrator drug and victim drug. The table provides information on the Korean allele frequency (wild type allele and variant allele) of SNPs related to the drugs. Furthermore, it contains information on the clinical impacts of the variant that are expected, thereby enabling clinicians to get useful insights into how often side effects from DDI will occur. The application also helps identify alternative drugs when the fold change is more significant than expected. This table provides the fold change for alternative drug pairs. The suggestion for an alternative pair of drugs was proposed as a result of selecting drugs with the same 4th ATC level of perpetrator/victim drug.

## Discussion

A machine learning model was proposed for the quantitative prediction of DDIs with high accuracy while constructing a highly-reliable PK-DDI DB. The PK-DDIP model satisfactorily predicted PK parameter fold changes when a perpetrator and a victim drug were simultaneously administered. The in-sample mean-squared error was 0.2494 and the RMSE of 5-fold cross-validation was 0.5959. Traditionally, a drug interaction between two drugs was determined by conducting clinical trials or designing a physiologically based pharmacokinetic (PBPK) model. It was impossible to perform drug interaction tests for all possible drug pairs. Our machine learning approach to predict PK parameter fold change is not constrained to a given gene list and thus improves model flexibility. It is expected that this model will enable the evaluation of potential interactions before performing human-based trials, which will significantly save time and cost. Furthermore, it will be possible to use machine learning techniques in evaluating DDIs for new drug approval.

One of the strengths of this study is that reliable data sources were manually collected and utilized. There are several well-known DBs widely used for developing models to predict DDIs, including DrugBank^44^, Kyoto Encyclopedia of Genes and Genomes^45^, NDF-RT^46^, and BioSNAP^47^ data with positive DDIs used for classification prediction^8–11,13–15,17–25,29,31,32,34–38,40,43,48^. SemMedDB contains DDI information extracted from PubMed^49^. TWOSIDES DB provides PD DDI information that utilizes DDI side effects data from the FDA adverse event reporting system^50^. SemMedDB and TWOSIDES have also been commonly used for classification prediction^12,15,16,20,23,27,28,30,33,41,42,48^. For the DeepDDI DB presented by Ryu et al.,^36^ DDIs are classified into 86 types by processing DDI information provided by DrugBank. The DeepDDI DB has been widely used in the development of multi-classification task models^20,28,30,39,41,42,51^. Most DDI information in these DBs often contains a large amount of data that have not been tested in actual clinical trials, only providing speculative information based on each drugs’ metabolic information or mechanical characteristics. For this reason, there is no information that includes drug pairs without an interaction (a true negative dataset). The PK-DDI DB was constructed from reliable FDA labels, whose data was based on actual clinical trials. Furthermore, the PK-DDI DB contains negative DDI pairs where DDIs did not occur and is the DB containing PK parameter change information. We hope this freely available PK-DDI DB will be widely used for various DDIs prediction studies to increase model performance. A more accurate and predictive model may be implemented if PK-DDI DB updated information is continuously collected. Another strength is that this PK-DDIP model has been externally validated by comparison with newly updated FDA labels. Furthermore, the results were confirmed with the validation of real-world patients taking drugs.

Quantitative prediction of drug exposure has usually been performed by using a PBPK model^52–55^. However, the PBPK method has poor versatility because it predicts a method limited to a specific drug or enzymes. Additionally, it takes a lot of time and technical expertise to construct a specific PBPK-based DDI model. The PK-DDIP model performed quantitative DDI prediction for all drugs with better performance for all-available drugs. In PBPK model studies, Waters et al. have evaluated a CYP450-mediated DDI prediction model for therapies used in the oncology setting and obtain RMSE values of 0.24-4.67 (combined model) and 0.20-2.45 (liver only model)^52^. Another study developed an in-vivo mechanistic static model (IMSM) with an RMSE range of 1.48-5.65^53^. Tod et al. show that their IMSM model predicts the proportion of predictions within 0.67- to 1.5-fold (corresponding to the *Case 1-2*) and within 0.5- to 2-fold (corresponding to the *Case 1-3*) as 79% and 93%, respectively (for DDIs mediated by P-glycoprotein and CYP3A4 only)^54^. In another study, the proportions are 90% and 99%, respectively (for DDIs mediated by organic anion transporting polypeptides, breast cancer resistance protein, and CYP2C8 only)^55^. In the PK-DDIP model, the RMSE value was 0.596, similar or lower than that in the PBPK models. The PK-DDIP model showed prediction proportions of 86.7% and 94.8% in *Case 1-2* and *Case 1-3*, respectively, and this result was not limited to specific DDI types.

Using the PK-DDI DB, quantitative AUC fold changes were predicted. Many studies have used a graph neural network to capture molecular structural features of drug pairs that generate DDIs^20,30,42,51^ and there has been an attempt to express the overall drug network by the occurrence of DDIs, and then extract the properties that cause interactions^41^. However, there is a fundamental problem with these attempts: they do not understand the underlying mechanisms of why DDIs occur. Although not used in this study, drugs have numerous properties other than their molecular structure; for example, properties, such as indication, adverse effects, molecular weight, and signaling pathway do not depend solely on the molecular structure. Therefore, it may be stated that a drug is an abstract concept that encompasses these characteristics and results. Unfortunately, there is no obvious data explaining why DDIs occur. Therefore, it is essential to use as many features as possible to preserve important information that might be missed, and at the same time, to include features that are expected to have the most significant impact on DDIs. In that respect, the relationship between the substrate, inhibitor, and inducer was analyzed in the description of fold changes in the meta dataset. Using this information, it was confirmed that interaction occurrence was very clearly divided according to the type of action and the AUC fold change was predicted with high accuracy.

However, there were some cases where an exception to this rule occurred, and the prediction failed. Rosuvastatin and fluconazole share cytochrome P450 2C9 and act as an enzyme-substrate and an enzyme inhibitor, respectively^56,57^. From the model, it would be expected that fluconazole would increase the rosuvastatin AUC fold change; the model predicted the AUC fold change to be 1.79. However, the rosuvastatin AUC fold change by fluconazole is 1.1 on the label^58^. In terms of quantitative modeling, it cannot be assumed that all enzymes cause an interaction, so it may be estimated that cytochrome P450 2C9 induces a wimpy interaction. Alternatively, cytochrome P450 2C9 mediates an interaction, but the effect is very insignificant, and it is speculated that some reaction between rosuvastatin and fluconazole inhibits the interaction. Another prediction error between ramelteon and fluvoxamine was caused by not having high enough expectations. These two drugs share a total of four CYP enzymes (1A2, 2C19, 2C9, and 3A4)^59,60^. Since the victim drug has all of these enzymes as a substrate and all of the perpetrator drugs have inhibitors, it was inferred that fluvoxamine would greatly raise the ramelteon AUC fold change. The model also expected a high ramelteon AUC fold change at 3.51. Nevertheless, the ramelteon AUC fold change by fluvoxamine is 190 on the label^61^, which is exceptionally high. Likewise, it was speculated that there were some other reactions or causes that need to be additionally considered. If many drug pairs share various enzymes or if an unknown influence is identified by adding various drug features to the features used for modeling, it will be possible to improve irregular pairs. However, this must be considered very carefully, as dimensionality is deeply bound by quantitative modeling.

It was not the goal for this model to be used for individual-level patient care. The PK-DDIP model was developed based on the PK-DDI DB from the FDA drug label. The PK fold change in the newly updated FDA label was predicted with high accuracy. However, the recommendation in the FDA drug label rely on a mean population recommendation, sometimes performed in healthy volunteers whom do not always reflect patient characteristics. Furthermore, tacrolimus is characterized by a wide range of inter-individual variability in its bioavailability^62^. Various clinical conditions have also been reported to affect tacrolimus PK, such as concomitant drugs, genotypes (e.g., SNPs), diet, and clinical values^63,64^. For this reason, the patients pool observed in a tertiary hospital clinical data warehouse (CDW) could be quite different from the ideal patient group, which might have caused the differences in predicted AUC fold change and the observed fold change for actual patients. Therefore, caution should be exercised when applying this model directly to a patient. Instead, we could reinforce therapeutic drug monitoring (TDM) of narrow therapeutic index (NTI) drugs such as tacrolimus after adding a perpetrator drug which is predicted to have a significant effect on the NTI drug.

Despite the several strengths, this study also has several important limitations. First, the DDI information was collected under specific controlled conditions in clinical trials. Different doses or usages usually result in different DDI results^65^. For instance, the effect of rifampin on the atorvastatin differs depending on the timing of administration and the duration of concurrent use^66^. Steady-state rifampin markedly decreases exposure when administration is separated, but slightly increases exposure when both drugs are given simultaneously; whereas, single-dose rifampin markedly increases exposure. However, it is not possible to predict different results when these conditions change; thus, the model only provides a rough average estimate. Second, the purpose of the study was to quantitatively predict the PK parameter fold change. The PK parameter fold change does not always result in a PD change or a side-effect occurrence. Therefore, another study might be needed to reveal the correlation between PK-PD. Lastly, the PK-DDIP model showed a poor performance in predicting extreme changes in AUC, reinforced TDM in the case of using a strong perpetrator drug is needed.

In conclusion, a PK-DDIP model was developed for the quantitative prediction of DDIs while constructing a PK-DDI DB. It is expected that many future studies will be conducted using the PK-DDIP model and PK-DDI DB. Further research is needed to elucidate the specific mechanisms of DDIs and improve model limitations.

## Methods

### DDI information data source

FDA drug labels were used to build a reliable DDI dataset as a training and validation dataset. The data were considered to be reliable in the following cases: 1) results of clinical trials performed on human subjects, 2) results using PBPK models. DDI information that has not been tested in actual clinical trials, only providing speculative information and not having any apparent fold change was not considered reliable. DDI information was collected using the DailyMed website (https://dailymed.nlm.nih.gov/dailymed/), which contains information on insert papers of drugs approved by the FDA. Information was extracted from 38,711 FDA drug labels (accessed date: May 2020), and the extracted items included: perpetrator drug, victim drug, and the AUC fold change. The perpetrator drug was a drug that caused a DDI, and a victim drug was affected by the interaction. If the perpetrator was reported to not affect the victim drug AUC, the AUC fold change value was 1. If the fold change had a ratio value other than 1, it meant that the ratio of the victim drug AUC with a perpetrator drug was divided by the one without the perpetrator drug. DDI information was manually obtained from sentences, figures, and tables in the clinical pharmacology and drug interaction sections in the drug label and collected by ten trained researchers. All data were reviewed twice by HJ and JS. The data were further processed based on principles discussed by the researchers to ensure data consistency.

### Data preprocessing

To determine the fold change of PK parameters in DDIs required for learning, the following data pre-processing standard operation procedure was defined as below. a) Absent a fold change in AUC: Cases were excluded that suggested an interaction between a drug class and a specific drug without presenting an apparent fold change (e.g.: ‘Antacids reduce the AUC of drug A’). b) Various dose and dosing times: If there were different AUC fold change values according to different dosing times, the AUC fold change values with simultaneous dosing times were selected. If there were different values according to different drug doses, results using the standard dose were selected. However, if several doses were standard, the geometric mean of the reported AUC fold changes was used. c) Interaction among three or more drugs: If drug A and drug B acted as perpetrator drugs and drug C acted as a victim drug, the features of drug A and drug B were combined and a new drug entity was assigned.

### Feature extraction

All drug features used in model development were obtained from the March 2021 version of DrugBank^44^. The most important feature was the binding relationship between the drug and the target/enzyme/carrier/transporter. This data was combined with information about polypeptide targets, enzymes, carriers, or transporters that ultimately were used to speculate which polypeptides might be involved in physiological effects and mechanisms of action. Polypeptides were identified using the Universal Protein Resource (UniProt) identifier. The gene name of a polypeptide, such as prostaglandin-endoperoxide synthase 1 (*PTGS1*), did not always have a one-to-one correspondence to the UniProt ID. If so, the gene name was manually matched. The second most important feature is its pharmacodynamics. The DrugBank defines the following 11 types of PD for drug pairs. We classified a drug pair as 11 types of PD action; otherwise, it was classified as negative PD (decrease adverse effects, decrease dynamics, decrease specific adverse effects, decrease specific effects, decrease therapeutic efficacy, increase adverse effects, increase dynamics, increase risk of hypersensitivity, increase specific adverse effects, increase specific effects, increase therapeutic efficacy).

### Feature vector encoding

The structure of a general sentence describing DDI was reflected when expressing the relationship between perpetrator and victim drugs. For example, DDIs are often described as follows: ‘The metabolism of silodosin can be decreased when combined with luliconazole^67^. Silodosin undergoes extensive metabolism via oxidative pathways mediated by CYP3A4. Potent CYP3A4 inhibitors may interfere with silodosin metabolism, resulting in increased serum concentrations of the drug and an elevated risk for developing drug-related adverse effects. Co-administration of 8 mg of silodosin and 400 mg of ketoconazole led to a 3.2-fold increase in AUC of silodosin^68^.’ As demonstrated in the description, CYP3A4 mediated the DDI between the perpetrator drug luliconazole and the victim drug silodosin, leading to a moderate effect on the increase of silodosin AUC. Furthermore, there is a substantial interplay between PK and the PD^69^. There is a PD interaction between luliconazole and silodosin, where the resulting drug concentration of silodosin at the site of action ultimately contributes to the PD response. The label DDI data were arranged where the perpetrator drug and the victim drug were centered, and various polypeptides/PD interactions were radially linked. Assuming that DDIs occurred according to the relationship of polypeptides and PD interactions linked to each drug, the primary information was summarized as the following four types.

- P-1 The linkage between each drug and polypeptide (e.g., related polypeptide in each perpetrator or victim drug)
- P-2 Types of polypeptides belonging to the drug (e.g., target, enzyme, carrier, or transporter)
- P-3 How the drugs affected each other’s polypeptides (e.g., inhibitor, inducer, or substrate)
- P-4 Types of PD action

Therefore, a polypeptide-PD-drug-type (PPDT) tokenization was proposed that as much as possible expressed DDI flexibly while reflecting the causal relationship. PPDT tokenization combined polypeptide, PD interaction, drug, and type into one word. For example, silodosin has {ATP binding cassette subfamily B member 1 [ABCB1], ABCB4, aldo-keto reductase family 1 member A1 [AKR1A1], aldehyde dehydrogenase 2 family member [ALDH2], CYP3A4, and UDP glucuronosyltransferase family 2 member B7 [UGT2B7]} as substrates and {adrenoceptor alpha 1A [ADRA1A], ADRA1B, and ADRA1D} as inhibitors^67^. However, luliconazole has {CYP2C19, CYP3A4, and lanosterol 14-alpha-demethylase [ERG11]} as inhibitors^70^. Therefore, the DDI of silodosin and luliconazole would be 13 tokens, including “CYP3A4PDinh CYP2C19PDinh ERG11PDinh CYP3A4VDsub UGT2B7VDsub ALDH2VDsub ADRA1AVDinh ADRA1BVDinh ADRA1DVDinh ABCB1VDsub ABCB4VDsub AKR1A1VDsub decrease_dynamics” (PD, perpetrator drug; VD, victim drug; inh, inhibitor; sub, substrate; decrease_dynamics. pharmacodynamic interaction type). Each token contained information on the relationship between each drug and polypeptide (P-1). The second information type, the type of polypeptide belonging to each drug, was reflected (P-2). The third information type included how the drugs affected each other’s polypeptides (P-3). The last information type included whether there is PD action or not (P-4). The extracted DDI information was PPDT-tokenized to obtain one document with 3,627 sentences with 2,830 unique tokens. After that, each DDI was encoded into a 2,830-dimensional vector through normalization using Term Frequency–Inverse Document Frequency (tf-idf).

### PK-DDIP model development

The AUC was selected as a proper PK parameter for prediction because various PK studies frequently rely on PK measures, such as the AUC to assess the extent of systemic exposure^71^. Therefore, the PK-DDIP model focused on predicting the effect of the perpetrator drug on the victim drug AUC. Of the extracted DDI information, 3627 pairs with feature information were used for training and tested. Log2 was used to transform the fold change to relax the distribution of values and then it was used for training. The regression bagged (bootstrap aggregation) trees method, which had the best performance, was used as the application model. The data characteristics needed to learn were as follows: First, it had a high dimension compared to the size of the data. Second, there were outliers on a specific range (strong increase/decrease). Small trees in bagged trees increased the level of decision-making. Therefore, an average performance improvement was expected because the abrupt change in variance by outliers did not significantly impair the tree bias. In addition, the lack of data was partially resolved through bootstrap aggregation, which repeatedly selected sample data. The tree consisted of 615 branches, and there were 308 nodes for which fold change values were determined. According to a decision rule, a branch was responsible for moving instances to the next level. If there was no branch in the node, the instance that arrived at the node had the fold change suggested by the node. If one DDI was predicted, it was decided as one fold change out of 308 values. The results of many decision trees were combined because individual decision trees tended to overfit. Therefore, the tree used bootstrap data samples to grow a decision tree from the ensemble. This process enabled an increase in the number of samples to 3627, which was small compared to 2830 dimensions. The number of trained learners in the ensemble was 30.

### Predictive model evaluation

Model evaluation was performed by calculating the difference between the fold change value presented in the label and the predicted value as the RMSE. The model was developed to have the lowest RMSE value. Further evaluation of model performance was conducted in two ways. First, DDIs were classified into several classes according to strength and whether the model predicted it correctly was evaluated using the modified FDA’s classification criteria^7^ as follows:

- A strong perpetrator drug increased the AUC of a victim drug ≥ 5-fold.
- A moderate perpetrator drug increased the AUC of a victim drug by ≥ 2- to <5-fold.
- A weak perpetrator drug increased the AUC of a victim drug by ≥ 1.25- to <2-fold.
- A strong perpetrator drug decreased the AUC of a victim drug by ≥ 80 percent.
- A moderate perpetrator drug decreased the AUC of a victim drug by ≥ 50 to <80 percent.
- A weak perpetrator drug decreased the AUC of a victim drug by ≥ 20 to <50 percent.

Furthermore, the fold change between 0.8- and 1.25-fold, which did not belong to any of the criteria, was defined as negative. Another model evaluation examined whether the predicted value was located within the pre-defined range. The numerical values were evaluated according to the following criteria, where *FC*_*lab*_ is the label AUC fold change, *FC*_*pre*_ is the predicted AUC fold change, *Class*_*lab*_ is the label class, and *Class*_*pre*_ is the predicted class.

- Case 1-1: {0.8 × *FC*_*lab*_ ≤ *FC*_*pre*_} ∧ {1.25 × *FC*_*lab*_ ≥ *FC*_*pre*_}
- Case 1-2: {0.67 × *FC*_*lab*_ ≤ *FC*_*pre*_} ∧{1.5 × *FC*_*lab*_ ≥ *FC*_*pre*_}
- Case 1-3: {0.5 × *FC*_*lab*_ ≤ *FC*_*pre*_} ∧{2 × *FC*_*lab*_ ≥ *FC*_*pre*_}
- Case 2: Class_lab_ = Class_pre_
- Case 3: Case 1-1 ∨ Case 2

The fold change uses the equation FC = (victim drug AUC in the presence of perpetrator)/(victim drug AUC in the absence of the test perpetrator). The FCs in decreased fold change of victim drug AUC are distributed between (0, 1), while the FCs in increased fold change of victim drug AUC are distributed between (1, ∞). Therefore, to compare the appropriate FC ranges, the values were compared by taking the inverse of the decreased fold change of victim drug AUC in DDIs in the (0, 1) interval. The interval range [0.8, 1.25] of Case 1-1 refers to the negative interval of the FDA, and the criteria [0.67, 1.5] and [0.5, 2] is used refer to the criteria in the PK DDI prediction study using a PBPK model^55^.

### Predictive model external validation

External validation of the predictive model was performed in two ways. First, newly updated FDA labels containing the DDI information section were collected using the ‘Drug Safety-related Labeling Changes’ search platform (https://www.accessdata.fda.gov/scripts/cder/safetylabelingchanges/) (assessed date: Jul 2021). The date range was set after the learning data collection period (after May 2020). Among them, label information that reported AUC changes of victim drugs were extracted and compared to the results predicted by the PK-DDIP model. The prediction results were defined as ‘good’ if AUC fold changes of the PK-DDIP model prediction were between 0.8- and 1.25-fold change of the newly updated drug label. If the only classification of whether the AUC fold change was >1 (or <1) was successful (meaning that the PK-DDIP model correctly predicted increase/decrease in AUC of victim drug), then the prediction results were considered ‘moderate’.

Another validation of the PK-DDIP model was performed by examining whether the prediction results of the PK-DDIP model were clinically observed in patients. The main object of this retrospective real-world study was to determine if there was a change in the blood concentration of the victim drug before and after taking the perpetrator drug.

Tacrolimus was selected as a victim drug for validation. Tacrolimus is an immunosuppressant drug widely used in most organ transplants, and its concentration is measured at regular intervals under a TDM system in a tertiary hospital^72^. For the list of perpetrator drugs, 18 drugs were selected by the research team among drugs predicted to affect the tacrolimus blood concentration. The study population included all patients who had been treated with tacrolimus using a CDW at a tertiary hospital between 2001 and 2021. The tacrolimus trough blood concentration is generally used as a simplified marker of drug exposure, and this correlates well with the AUC^73,74^. Therefore, the measured tacrolimus trough blood level was collected just before and every 1–2 days after initiating perpetrator drugs for seven days^75^. Because the physician applied adjusted doses of tacrolimus in response to changed blood levels, the tacrolimus concentration/dose (C/D) ratio was calculated seven days after initiating perpetrator drugs when the tacrolimus blood levels had stabilized. The changed C/D ratio at day seven was divided by the one at day 0, and the obtained value was compared with the prediction value from the PK-DDIP model. Approval from the Institutional Review Board at Seoul National University hospital was obtained prior to collecting and analyzing the data (IRB No. 2107-233-1240). Written informed consent was not required for CDW-based studies using anonymized data.

### Standalone application

The following additional information for an all-around understanding of the user’s DDI was provided in a standalone application. The application provides SNP actions from DrugBank and its allele frequency in Koreans obtained from KRGDB^76^ SNPs associated with drug activity or metabolism can affect pharmacological activity. The SNP information of each perpetrator or victim drug can be considered for dose escalation or therapy change when administering the drug to a patient. The application additionally recommends alternative drugs whose ATC 4th levels are same with perpetrator or victim drugs. The hierarchical structure of ATC can be found at https://www.whocc.no.

### Reporting summary

Further information on research design is available in the Nature Research Reporting Summary linked to this article.

## Supplementary information

Supplementary information

Reporting Summary

## Data Availability

The PK-DDI DB is freely available from Github repository: https://github.com/harryscpt/pk-ddip. The standalone application can be operated by connecting to https://pk-ddi.snu.ac.kr/en. Access to the standalone application website is possible after obtaining an access code through a request to the corresponding author, JO.

## Code availability

The source code used to develop our PK-DDIP model is available from Github repository: https://github.com/harryscpt/pk-ddip.
