# Supplementary material for: Machine learning-based quantitative prediction of drug exposure in drug-drug interactions using drug label information
Source: NPJ Digit Med. 2022 Jul 11;5:88. doi: 10.1038/s41746-022-00639-0 (PMC9273620; doi:10.1038/s41746-022-00639-0)
Supplement: Supplementary file 1 — Supplementary information [file 41746_2022_639_MOESM1_ESM.pdf]

Machine learning-based quantitative prediction of drug exposure in drug-drug interactions using drug label information

Ha Young Jang, Jihyeon Song, Jae Hyun Kim, Howard Lee, In-Wha Kim, Bongki Moon, and Jung Mi Oh

SUPPLEMENTARY INFORMATION

Supplementary Figure 1. Flow chart for drug-drug interaction (DDI) database

Supplementary Figure 2. Flow chart for collecting drug concentrations of real-patients in tertiary-hospital for evaluating prediction results.

Supplementary Table 1. Obtained root-mean-squared error (RMSE) value for each model

Supplementary Table 2. Median percent error of the predicted fold change from the label in each drug-drug interaction classes according to strength

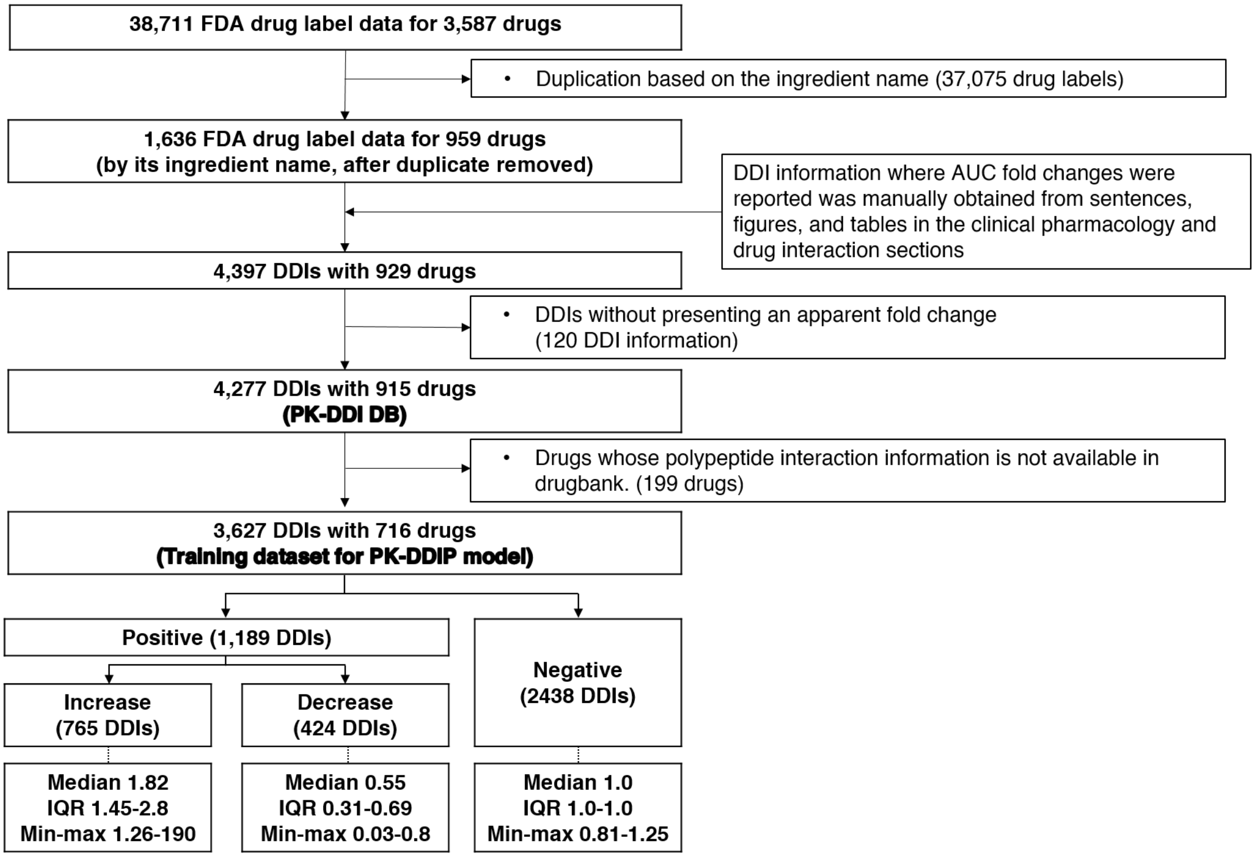

Supplementary Figure 1. Flow chart for drug-drug interaction (DDI) database. AUC, area under the time-concentration curve; DDI, drug-drug interaction; FDA, Food and Drug Administration; IQR, interquartile range; PK-DDI DB, pharmacokinetic DDI database; PK-DDIP, PK-DDI prediction.

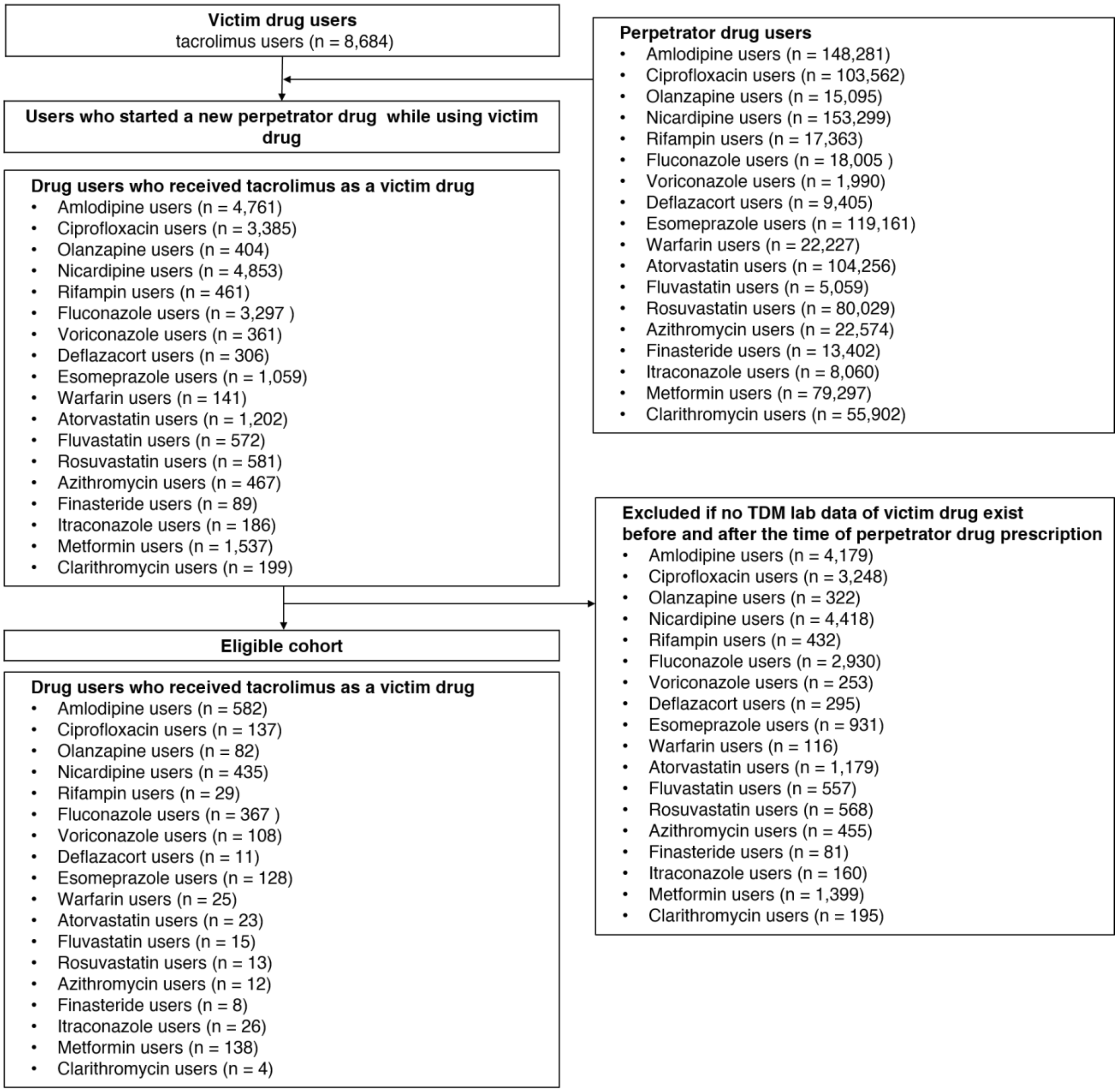

Supplementary Figure 2. Flow chart for collecting drug concentrations of real-patients in tertiary-hospital for evaluating prediction results. TDM, therapeutic drug monitoring.

Supplementary Table 1. Obtained root-mean-squared error (RMSE) value for each model

	Model	RMSE
Linear Regression Models	Linear	1.042
	Robust Linear	1.193
Regression Trees	Fine Tree	0.678
	Medium Tree	0.638
	Coarse Tree	0.650
	Fine Gaussian SVM	0.787
	Medium Gaussian SVM	0.713
	Coarse Gaussian SVM	0.810
Gaussian Process Regression Models	Rational Quadratic	0.637
	Squared Exponential	0.645
	Matern 5/2	0.642
	Exponential	0.638
Ensembles of Trees	Boosted Trees	0.615
	**Bagged Trees**	**0.596**

SVM, support vector machine

Supplementary Table 2. Median percent error of the predicted fold change from the label in each drug-drug interaction classes according to strength

Label	Median percent error
Strong Increased	65.45%
Moderate Increased	31.25%
Weak Increased	17.56%
Negative	4.38%
Weak Decreased	30.30%
Moderate Decreased	38.90%
Strong Decreased	90.34%
