# Supplementary material for: Machine learning-based quantitative prediction of drug exposure in drug-drug interactions using drug label information
Source: NPJ Digit Med. 2022 Jul 11;5:88. doi: 10.1038/s41746-022-00639-0 (PMC9273620; doi:10.1038/s41746-022-00639-0)
Supplement: Supplementary file 2 — Reporting Summary [file 41746_2022_639_MOESM2_ESM.pdf]

nature portfolio

Corresponding author(s): Jung Mi Oh

Last updated by author(s): Jun 14, 2022

Reporting Summary

Nature Portfolio wishes to improve the reproducibility of the work that we publish. This form provides structure for consistency and transparency in reporting. For further information on Nature Portfolio policies, see our Editorial Policies and the Editorial Policy Checklist.

Statistics

For all statistical analyses, confirm that the following items are present in the figure legend, table legend, main text, or Methods section.

n/a	Confirmed	
☐	☒	The exact sample size (*n*) for each experimental group/condition, given as a discrete number and unit of measurement
☐	☒	A statement on whether measurements were taken from distinct samples or whether the same sample was measured repeatedly
☐	☒	The statistical test(s) used AND whether they are one- or two-sided<br>*Only common tests should be described solely by name; describe more complex techniques in the Methods section.*
☐	☒	A description of all covariates tested
☒	☐	A description of any assumptions or corrections, such as tests of normality and adjustment for multiple comparisons
☐	☒	A full description of the statistical parameters including central tendency (e.g. means) or other basic estimates (e.g. regression coefficient) AND variation (e.g. standard deviation) or associated estimates of uncertainty (e.g. confidence intervals)
☒	☐	For null hypothesis testing, the test statistic (e.g. *F*, *t*, *r*) with confidence intervals, effect sizes, degrees of freedom and *P* value noted<br>*Give P values as exact values whenever suitable.*
☒	☐	For Bayesian analysis, information on the choice of priors and Markov chain Monte Carlo settings
☐	☒	For hierarchical and complex designs, identification of the appropriate level for tests and full reporting of outcomes
☐	☒	Estimates of effect sizes (e.g. Cohen's *d*, Pearson's *r*), indicating how they were calculated

Our web collection on statistics for biologists contains articles on many of the points above.

Software and code

Policy information about availability of computer code

Data collection	No software was used for data collection.
Data analysis	The machine learning analysis was performed through matlab software, and the code used is available in the Github repository (https://github.com/harryscpt/pk-ddip).

For manuscripts utilizing custom algorithms or software that are central to the research but not yet described in published literature, software must be made available to editors and reviewers. We strongly encourage code deposition in a community repository (e.g. GitHub). See the Nature Portfolio guidelines for submitting code & software for further information.

Data

Policy information about availability of data

All manuscripts must include a data availability statement. This statement should provide the following information, where applicable:

- Accession codes, unique identifiers, or web links for publicly available datasets
- A description of any restrictions on data availability
- For clinical datasets or third party data, please ensure that the statement adheres to our policy

The PK-DDI DB is freely available from Github repository: https://github.com/harryscpt/pk-ddip.

Field-specific reporting

Please select the one below that is the best fit for your research. If you are not sure, read the appropriate sections before making your selection.

☒ Life sciences ☐ Behavioural & social sciences ☐ Ecological, evolutionary & environmental sciences

For a reference copy of the document with all sections, see nature.com/documents/nr-reporting-summary-flat.pdf

Life sciences study design

All studies must disclose on these points even when the disclosure is negative.

Sample size	Since all available data were collected, the sample size required for analysis was not calculated. Sufficient prediction accuracy was confirmed through the collected data.
Data exclusions	We excluded patients if no therapeutic drug monitoring lab data of victim drug exist before and after the time of perpetrator drug prescription. The exclusion criteria were reviewed by the IRB review.
Replication	All analyzes were performed with the database and code, and the reproducibility of the results was confirmed through repeated analysis.
Randomization	Part of this study was conducted as a retrospective cohort study, and there is no separate experimental group/control group. Therefore, randomization was not performed.
Blinding	All patient information in the clinical data warehouse was anonymized.

Reporting for specific materials, systems and methods

We require information from authors about some types of materials, experimental systems and methods used in many studies. Here, indicate whether each material, system or method listed is relevant to your study. If you are not sure if a list item applies to your research, read the appropriate section before selecting a response.

Materials & experimental systems

n/a	Involved in the study	
☒	☐	Antibodies
☒	☐	Eukaryotic cell lines
☒	☐	Palaeontology and archaeology
☒	☐	Animals and other organisms
☐	☒	Human research participants
☒	☐	Clinical data
☒	☐	Dual use research of concern

Methods

n/a	Involved in the study	
☒	☐	ChIP-seq
☒	☐	Flow cytometry
☒	☐	MRI-based neuroimaging

Human research participants

Policy information about studies involving human research participants

Population characteristics	The study population included all patients who had been treated with tacrolimus using a clinical data warehouse (CDW) at a tertiary hospital between 2001 and 2021.
Recruitment	The study population included all patients who had been treated with tacrolimus using a clinical data warehouse (CDW) at a tertiary hospital between 2001 and 2021. We excluded patients if no therapeutic drug monitoring lab data of victim drug exist before and after the time of perpetrator drug prescription. Written informed consent was not required for CDW-based studies using anonymized data.
Ethics oversight	Institutional Review Board at Seoul National University hospital (IRB No. 2107-233-1240)

Note that full information on the approval of the study protocol must also be provided in the manuscript.
